# Scrotal Edema as a Rare Manifestation of Diabetic Ketoacidosis in the Setting of Hypoalbuminemia: A Case Report

**DOI:** 10.7759/cureus.92129

**Published:** 2025-09-12

**Authors:** Andrew D Nguyen, Kristian E Gunsalus, Nicholas Zulia, Michael Lapoint

**Affiliations:** 1 Medicine, Lake Erie College of Osteopathic Medicine, Erie, USA; 2 Medicine, Niagara Falls Memorial Medical Center, Niagara Falls, USA

**Keywords:** diabetic foot wound, diabetic ketoacidosis (dka), diabetic peripheral neuropathy (dpn), prerenal acute kidney injury, scrotal edema, severe diabetic neuropathy, staphylococcal cellulitis, types 2 diabetes

## Abstract

We present the case of a 64-year-old male with a history of type 2 diabetes mellitus and bilateral lower limb neuropathy who initially presented with fever, leukocytosis, and right lower leg cellulitis. Imaging revealed a retained diabetic needle with a surrounding abscess that was treated with debridement. Wound debridement cultures grew methicillin-resistant *Staphylococcus aureus*, and he was discharged on a course of oral linezolid. Three days following discharge, the patient was readmitted with hypotension, prerenal acute kidney injury (AKI), hypoalbuminemia, and hyperglycemia due to insulin nonadherence. Venous blood gas confirmed diabetic ketoacidosis (DKA). During hospitalization, the patient developed painless scrotal edema. The scrotal edema was treated with intravenous albumin and oral bumetanide. This edema was likely caused by generalized hypoalbuminemia from aggressive fluid resuscitation and insulin treatment, in conjunction with prerenal AKI. This case highlights scrotal edema as a rare sequela of DKA that should be considered after urgent causes are ruled out, especially in cases of chronic disease nonadherence and large-volume fluid therapy.

## Introduction

Scrotal edema is an uncommon finding with a broad differential diagnosis, including systemic, local, and iatrogenic etiologies. Most frequently, systemic causes include congestive heart failure, hepatic cirrhosis, nephrotic syndrome, and hypoalbuminemia, while local factors include infection, trauma, or lymphatic trauma. In the setting of recent diabetic ketoacidosis (DKA) and subsequent insulin therapy, the differential can be expanded to include fluid resuscitation and insulin-induced etiologies [1-8].

In the present case, the development of scrotal edema following DKA and insulin nonadherence was likely multifactorial. More specifically, aggressive intravenous fluid administration and insulin therapy likely contributed to generalized hypoalbuminemic edema, while prerenal acute kidney injury (AKI) may have dampened fluid clearance [4,8]. Regarding pathophysiology, some of these mechanisms are not well understood and are primarily supported by case reports and small case series, especially for insulin-induced edema.

Other common etiologies were considered and ruled out through clinical and laboratory evaluation. This case exemplifies the importance of a comprehensive diagnostic approach to the atypical finding of scrotal edema in patients with diabetes, especially in the context of guiding treatment and excluding serious etiologies.

## Case presentation

A 64-year-old male with a history of type 2 diabetes mellitus and bilateral lower limb neuropathy came in with a concern of hypertension and right lower leg edema with erythema and warmth. Initial vitals showed fever (38.8°C), tachycardia (115 beats/minute), and leukocytosis (30.2 × 10⁹/L with a left shift), suggestive of an infectious process.

**Figure 1:**
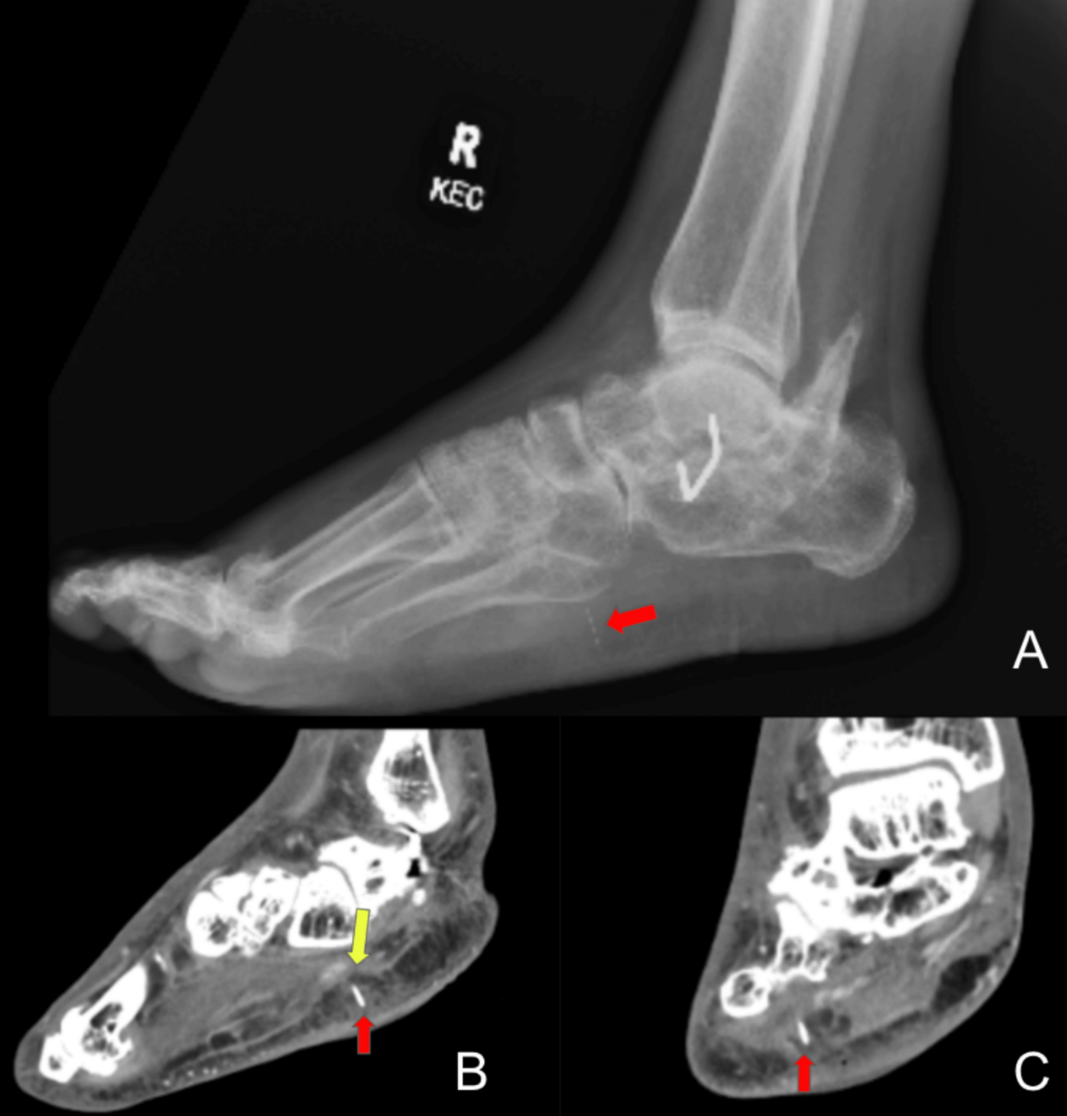
Lateral X-ray of the right foot (A), sagittal (B) and coronal (C) CT soft tissue window before wound debridement on first admission. A small foreign body, later found to be an insulin needle, measuring 1.3 cm in length, was noted on the plantar surface, 1 cm deep into the skin surface (red arrow). The surrounding area of the foreign body demonstrated soft tissue swelling and ectopic air (yellow arrow), suggestive of abscess formation.

**Figure 2:**
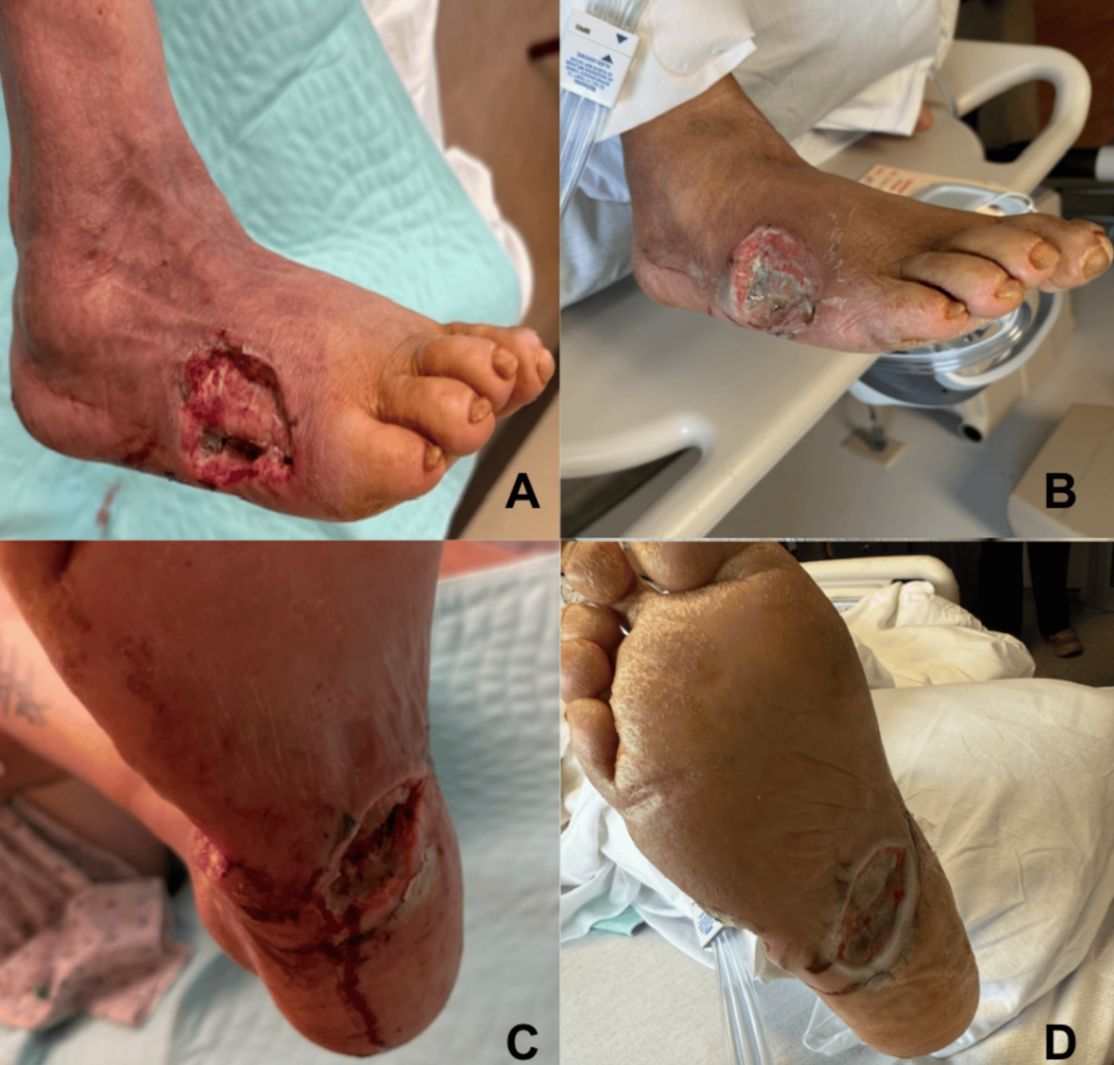
Photos of wound debridement postoperative day three (A and C) on first admission and postoperative day 13 (B and D) on second admission. Images demonstrate delayed healing by secondary intention with limited granulation tissue. Informed consent was obtained.

A plain radiograph performed shortly after admission revealed a 1.3 cm diabetic needle in the plantar surface of the right foot (Figure 1A). The follow-up CT scan subsequently revealed abscess formation surrounding the needle (Figures 1B, 1C). The patient underwent surgical debridement of the dorsal and plantar surfaces. Intraoperative cultures were obtained and revealed wound infection with methicillin-resistant *Staphylococcus aureus* (Figure 2). The patient was treated with oral linezolid and was discharged after a 10-day stay.

**Figure 3:**
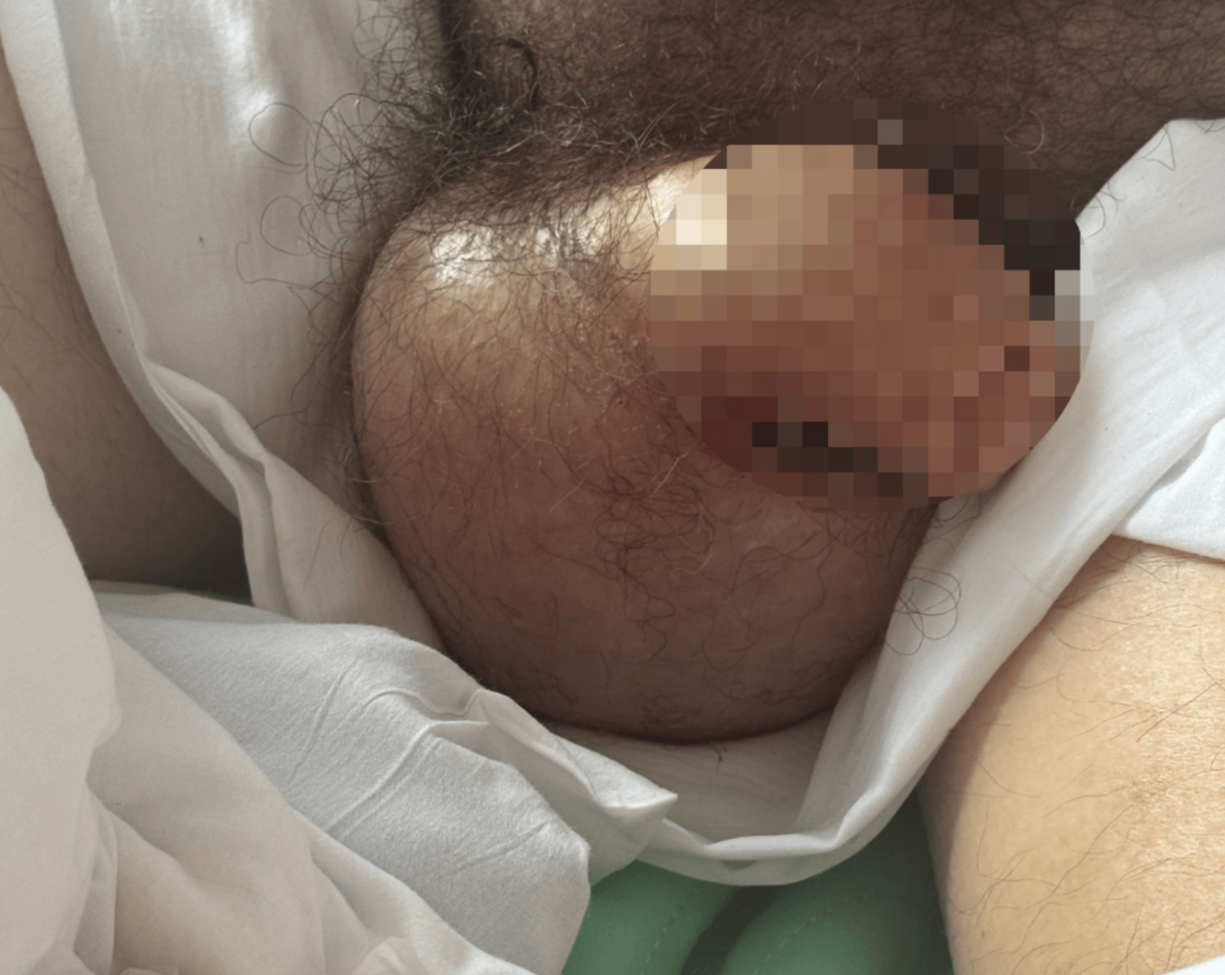
Scrotal edema that developed following diabetic ketoacidosis. The scrotum measured approximately 10 cm in diameter. Ultrasound revealed only edema without acute pathology. Informed consent was obtained from the patient for the publication of the image.

Three days post-discharge, the patient returned to the emergency department with the major complaint of dizziness and hyperglycemia following noncompliance with his basal bolus insulin regimen. He was hypotensive (95/55 mmHg) with a serum glucose of 1,001 mg/dL. Additionally, the patient presented with an elevated blood urea nitrogen (60 mg/dL) and creatinine (2.08 mg/dL), suggestive of a prerenal AKI. Additional labs on both admissions are shown in Table 1. Laboratory workup revealed hypoalbuminemia (albumin 2.4 g/dL), and venous blood gas findings confirmed DKA (Table 2). During hospitalization, the patient developed painless scrotal edema, which progressed even as his white blood cell count normalized (down to 8.8 × 10⁹/L) (Figure 3). His DKA was treated with intravenous normal saline at 1 L/hour, regular insulin infusion, and potassium repletion per protocol. Scrotal ultrasound revealed edema with minor bilateral wall thickening and no concern for vascular abnormality or varicocele. The edema was managed with 12.5 g IV 25% albumin BID and 1 mg oral bumetanide once a day until resolution.

**Table 1:** Additional lab values of the patient upon initial admission, discharge, and readmission. *: 1+ hemolysis of solution.

	Normal range	First admission	First discharge	Second admission
Sodium (mmol/L)	135–145	136	142	128
Potassium (mmol/L)	3.5–5.6	4.9	5.0*	6.2
Chloride (mmol/L)	96–110	102	111	96
CO₂/Bicarbonate (mmol/L)	20–32	26	25	6
Anion gap (mmol/L)	5–15	8	6	26
Glucose (mg/dL)	60–100	75	164	1,001
Blood urea nitrogen (mg/dL)	5–27	22	22	60
Creatinine (mg/dL)	0.4–1.4	1.25	1.13	2.08
Estimated glomerular filtration rate (mL/minute/1.73m²)	≥60	64	73	35
Calcium, total (mg/dL)	8.5–10.5	7.8	7.7	7.6
Total protein (g/dL)	5.8–7.8	4.9	4.6	5.1
Albumin (g/dL)	3.3–4.8	2.8	2.3	2.6
White blood cells count (×10³/µL)	4.0–10.5	30.2	11.8	16.5

**Table 2:** Venous blood gas values upon second admission to the emergency department.

	Normal tange	Patient’s values
pH	7.34–7.44	7.06
pCO₂ (mmHg)	35–45	14.4
pO₂ (mmHg)	75–100	102
HCO₃⁻ (mmol/L)	22–26	4.13

## Discussion

We present an unusual case, with our patient initially presenting with an incidental finding of a retained diabetic needle in the foot and ending with the development and treatment of scrotal edema following an episode of DKA and insulin nonadherence. Previous reports have only shown one similar case in which insulin therapy resulted in scrotal edema [7]. Scrotal edema is a clinical finding that has a broad differential and is often multifactorial. In the context of this case, the most likely causes are generalized hypoalbuminemic edema due to fluid resuscitation and insulin therapy.

To treat our patient’s DKA, he was given 1 L of normal saline per hour, followed by 1 L of D5 ½ normal saline per hour. Given this aggressive fluid resuscitation, it is possible that the albumin concentration was further reduced, resulting in a reduction in oncotic pressure and promoting fluid extravasation into peripheral spaces such as the scrotum [1,5,8]. This, in conjunction with our patient’s pre-renal AKI, likely contributed to a volume-overloaded state, increasing hydrostatic pressure and favoring third spacing.

Furthermore, previous literature has shown that for patients with few comorbidities, insulin therapy can result in an edematous state [3,6]. Although this pathophysiology has not been heavily explored, some studies have concluded that insulin administration can increase sodium reabsorption from the diluting segment of distal nephrons [2]. Given our patient’s history and lab values, it is likely that these factors played a role in the development of his scrotal edema.

Notably, the differential diagnosis for scrotal edema can also include hepatic cirrhosis, congestive heart failure, nephrotic syndrome, or infection. However, in the absence of elevated liver function tests, a recent unremarkable echocardiogram, trace proteinuria, and resolution of white cell count, it is unlikely that these etiologies are relevant. Finally, the absence of pain and lack of concerning ultrasound findings made urgent etiologies such as Fournier gangrene or testicular torsion unlikely. Thus, we suspect generalized hypoalbuminemic edema due to fluid resuscitation and insulin therapy.

## Conclusions

Painless scrotal edema is a relatively unique and alarming symptom that often causes great distress to patients despite its asymptomatic clinical course. While common causes of edema, such as heart failure and cirrhosis of the liver, should be considered and tested for, this case presentation highlights the importance of expanding into a broader differential diagnosis of a catabolic state, such as DKA, where hypoalbuminemia is likely present. It is also important to consider the systemic consequences of chronic nonadherence in diabetic patients. In patients with diabetes, continued nonadherence to medications and poor lifestyle choices can lead to further complications and difficulty maintaining metabolic and electrolyte homeostasis, causing a preventable second hospitalization. Ultimately, this case highlights a potential avenue for further exploration of fluid resuscitation and insulin edema.
